# Novel *HBB:*c.375_376delAC mutation in a Malay patient with HbE beta-thalassemia intermedia: A case report

**DOI:** 10.1097/MD.0000000000044817

**Published:** 2025-09-26

**Authors:** Nur Aisyah Aziz, Ernie Zuraida Ali, Mohd Khairul Nizam Mohd Khalid, Nursaedah Abdullah Aziz, Norodiyah Othman, Faidatul Syazlin Abdul Hamid, Syahzuwan Hassan, Ezalia Esa, Wan Rohani Wan Taib, Khairul Azhar Abu Bakar

**Affiliations:** a Hematology Unit, Cancer Research Center (CaRC), Institute for Medical Research (IMR), National Institute of Health (NIH), Ministry of Health (MOH), Shah Alam, Selangor, Malaysia; b Faculty of Health Science, University Sultan Zainal Abidin, Kuala Terengganu, Terengganu, Malaysia; c Inborn Error of Metabolism and Genetic Unit, Nutrition, Metabolism and Cardiovascular Research Centre (NMCRC), Institute for Medical Research (IMR), National Institute of Health (NIH), Ministry of Health (MOH), Shah Alam, Selangor, Malaysia; d Department of Medicine, Hospital Sultanah Abdul Halim, Ministry of Health (MOH), Sungai Petani, Kedah, Malaysia.

**Keywords:** *HBB* gene, HbE beta-thalassemia, Malaysia, molecular diagnosis, novel frameshift mutation

## Abstract

**Rationale:**

Hemoglobin E beta-thalassemia (HbE-BT) is a prevalent and genetically diverse inherited hemoglobin disorder in Malaysia. While most cases are attributed to known genetic variants, discovering novel mutations is essential for understanding their wide-ranging clinical presentations and underlying genetic heterogeneity.

**Patient concerns:**

We present the case of a Malay male patient with an intermediate phenotype of HbE-BT whose clinical course was marked by a progression to a transfusion-dependent status, a change triggered by an acute febrile illness. The case highlights the complex challenges of long-term management, including a high risk of iron overload and the significant morbidity and mortality associated with postsplenectomy infections.

**Diagnoses:**

The patient was referred for a comprehensive DNA analysis of thalassemia syndromes. Full blood count showed hypochromic microcytic anemia (hemoglobin 7.90 g/dL, mean corpuscular volume 66.10 fL). Hemoglobin analysis via capillary electrophoresis revealed a characteristic pattern of HbA (49.1%), HbE (20.6%), and HbF (27.0%). Sanger sequencing subsequently confirmed a compound heterozygous state of HBB:c.79G>A (HbE) and a novel frameshift mutation at codon 124 of the beta-globin gene (HBB:c.375_376delAC). Predictive computational tools further supported the pathogenicity of this new variant, and its data have been submitted to the ITHANET database (IthaID: 4155).

**Interventions:**

Initially, the patient’s condition was managed with nutritional supplements and intermittent blood transfusions. Following an acute febrile episode at age 26, his clinical condition worsened, necessitating regular monthly blood transfusions to manage severe anemia. He also received subcutaneous chelation therapy to address severe hepatic iron overload. At age 36, he underwent a splenectomy to reduce his transfusion requirements.

**Outcomes:**

Despite these interventions, the patient passed away at the age of 37 from septic shock and multiorgan failure. This outcome occurred a year after his splenectomy and underscores the critical risk of overwhelming postsplenectomy infection, a significant complication in splenectomized thalassemia patients.

**Lessons:**

Identifying this novel compound heterozygosity contributes to our understanding of the genetic landscape of HbE-BT in Malaysia. This case reinforces the importance of comprehensive molecular analysis for accurate diagnosis, and it has important implications for improving long-term management and genetic counseling strategies for patients with thalassemia syndromes.

## 1. Introduction

Hemoglobin E beta-thalassemia (HbE-BT) is a prevalent inherited hemoglobin disorder in Southeast Asia, including Malaysia,^[1,2]^ characterized by wide-ranging genetic and clinical features. Confirming the diagnosis often necessitates molecular testing. Challenges such as limited access to testing and low public awareness frequently delay diagnosis, and the varied clinical severity of HbE-BT makes symptom-based diagnosis difficult. Moreover, clinical symptoms often overlap with other hemoglobinopathies, further complicating diagnosis efforts. Consequently, DNA analysis is essential for the proper management of HbE-BT, as it helps differentiate the disease and informs genetic counseling and prenatal diagnosis for at-risk families.

The HbE variant (*HBB*:c.79G>A) originates from a single nucleotide substitution (*G*AG > *A*AG) at the 26th codon of the beta-globin gene (*HBB*; NM_000518.5). This mutation creates an alternative splice donor site within exon 1 of the *HBB* gene, ultimately leading to nonsense-mediated mRNA decay.^[3,4]^ In both heterozygous and homozygous states, HbE is typically asymptomatic and mildly anemic.^[5]^ However, the biophysical stability of HbE is inherently lower than that of HbA and HbA_2,_ making it more vulnerable to oxidative stress in the red blood cells of HbE-BT patients.^[6]^ Therefore, individuals with compound heterozygous states of HbE and beta-thalassemia mutations exhibit a broad spectrum of clinical manifestations, ranging from severe transfusion-dependent forms to mild intermediate presentations.^[7]^

In Peninsular (West) Malaysia, Malays constitute the predominant ethnic group and account for the majority of HbE carriers, leading to the highest prevalence of HbE (5%) in the country.^[8]^ Beyond HbE, other common *HBB* variants among Malays include codon 41/42 (‐TTCT) [*HBB*:c.126_129delCTTT], IVSI-5 (G > C) [*HBB*:c.92 + 5G>C], and IVSI-1 (G > T) [*HBB*:c.92 + 1G>T].^[9]^ The advent of molecular diagnostics has significantly advanced the exploration of HbE-BT’s genetic diversity in Malaysia. Despite identifying common *HBB* variants, the range of rare or novel mutations causing variability in HbE-BT, especially in Malaysia, is not fully understood. This highlights the need for detailed molecular studies of individual cases, even for common disorders. Novel *HBB* mutations’ effects on mRNA, protein, and clinical outcomes often require extensive investigation through case reports and functional studies. A better understanding of genetic variations can improve screening strategies and the prevention plan. This report describes a novel frameshift mutation, *HBB*:c.375_376delAC, at codons 124/125, in a Malay patient with HbE-BT intermedia, in compound heterozygosity with HbE. The report aims to fill this knowledge gap by characterizing the mutation and its functional impact through predictive analyses. It also discusses the clinical implications of transfusion-dependent beta-thalassemia and infection risks postsplenectomy, offering insights into management challenges of HbE-BT.

## 2. Case presentation

A 29-year-old Malay male was referred to our laboratory for routine DNA analysis of thalassemia syndrome. The patient’s full blood count revealed hypochromic microcytic anemia, with hemoglobin (Hb) 7.90 g/dL, red blood cells 3.63 × 10^6^/μL, mean corpuscular volume 66.10 fL, and mean corpuscular hemoglobin 21.80 pg. Hemoglobin analysis, performed using automated capillary electrophoresis (Sebia), showed a low HbA of 49.1%, HbA_2_ at 3.3%, the presence of variant HbE at 20.6%, and an elevated HbF of 27.0%. These findings—significantly reduced Hb, mean corpuscular volume, and mean corpuscular hemoglobin, along with elevated HbF and the presence of HbE—led to a presumptive diagnosis of HbE-BT (Table 1) and prompted referral to our laboratory for DNA analysis of thalassemia syndrome. Although the patient claimed a thalassemia diagnosis was made when he was 10 years old, there was no documented evidence from the hemoglobin or DNA analysis that was found, nor were there records of blood transfusions before his 26th year at the treating hospital. This study was done following the 2024 Helsinki Declaration. Written informed consent was obtained from the patient for the genetic testing and publication of this case report. This study was reviewed and approved by the Medical Research and Ethical Committee (MREC), Ministry of Health, Malaysia (NMRR-18-3977-43849).

**Table 1:** Summary of clinical history, laboratory findings, and in silico analysis of *HBB*:c.375_376delAC genetic variant associated with the patient.

Parameters	Patient
Age (yr)/sex	29/Male
Hemoglobin	7.9 g/dL
Red blood cell (RBC)	3.63 × 10^6^/µL
Mean corpuscular volume (MCV)	66.1 fL
Mean corpuscular hemoglobin (MCH)	21.8 pg
Mean corpuscular hemoglobin concentration (MCHC)	32.9 g/dL
Red cell distribution width-CV (RDW-CV)	28.2 %
Splenectomy	Yes (at 36 years old)
Status	He passed away at 37 years old
Capillary electrophoresis	
HbA	49.1%
HbA_2_	3.3%
HbF	27.0%
HbE	20.6%
DNA analysis	
β-MARMS	β^E^/β
β-Sequencing	β^E^/β^Codon124_125delAC^
α-MGap	αα/αα
α-MARMS	αα/αα
δ-Sequencing	δ/δ
In silico analysis	
MutationTaster2021	Deleterious
Variant effect predictor (VEP)	High impact (disruptive)
Franklin	Likely pathogenic
VarSome	Likely pathogenic
FATHMM	Damaging (score: ‐3.21)

HbA = normal adult hemoglobin, HbA_2_ = minor adult hemoglobin, HbF = fetal hemoglobin, β-MARMS = beta gene multiplex amplification-refractory mutation system PCR, β-Sequencing = beta gene Sanger sequencing, α-MARMS = alpha gene multiplex amplification-refractory mutation system PCR, α-MGap = alpha gene multiplex gap PCR, β^E^ = variant at codon 26 (*G*AG > *A*AG) HbE of *HBB* gene (*HBB*:c.79G>A), β^Codon124/125delAC^ = frameshift variant at the junction of codon 124 and 125 (‐AC) of *HBB* gene (*HBB*:c.374_375delCA), αα/αα = normal allele of *HBA1/2* genes, δ/δ = normal allele of *HBD* gene.

### 2.1. Clinical course and management

The patient initially sought medical attention with a 3-day history of flu-like illness (fever, dry cough, and malaise). Upon clinical examination, thalassemia features with pallor of the conjunctivae, prominent frontal bossing, and palpable hepatosplenomegaly. Despite an initial Hb of 2.1 g/dL, the patient denied any symptoms of anemia (e.g., giddiness, palpitations, breathlessness, chest pain, or any bleeding tendencies). ECG also showed no significant changes to suggest anemia-induced angina. The patient was an active smoker and reported taking vitamin B complex supplements. He also reported receiving occasional blood transfusions whenever his hemoglobin fell below 8 g/dL, up until he was 17 years old. Following hospitalization for a high fever at age 26, the patient’s condition progressed, necessitating monthly blood transfusions within a year, and developed severe hepatic iron overload. According to the Malaysian Clinical Practice Guidelines for thalassemia management, the patient’s initial pre-transfusion hemoglobin level typically ranges from 2 to 5 g/dL. After 1 year of follow-up in 2014, the patient was unable to maintain the adequate hemoglobin target of 8 g/dL, thereby requiring monthly blood transfusions. Achieving and maintaining an adequate hemoglobin target is crucial for ensuring proper organ perfusion and for preventing cardiac complications such as cardiac compromise and high-output cardiac failure secondary to anemia. Nevertheless, the decision to transfuse must always be carefully weighed against the significant risk of iatrogenic iron overload.

**Figure 1.**
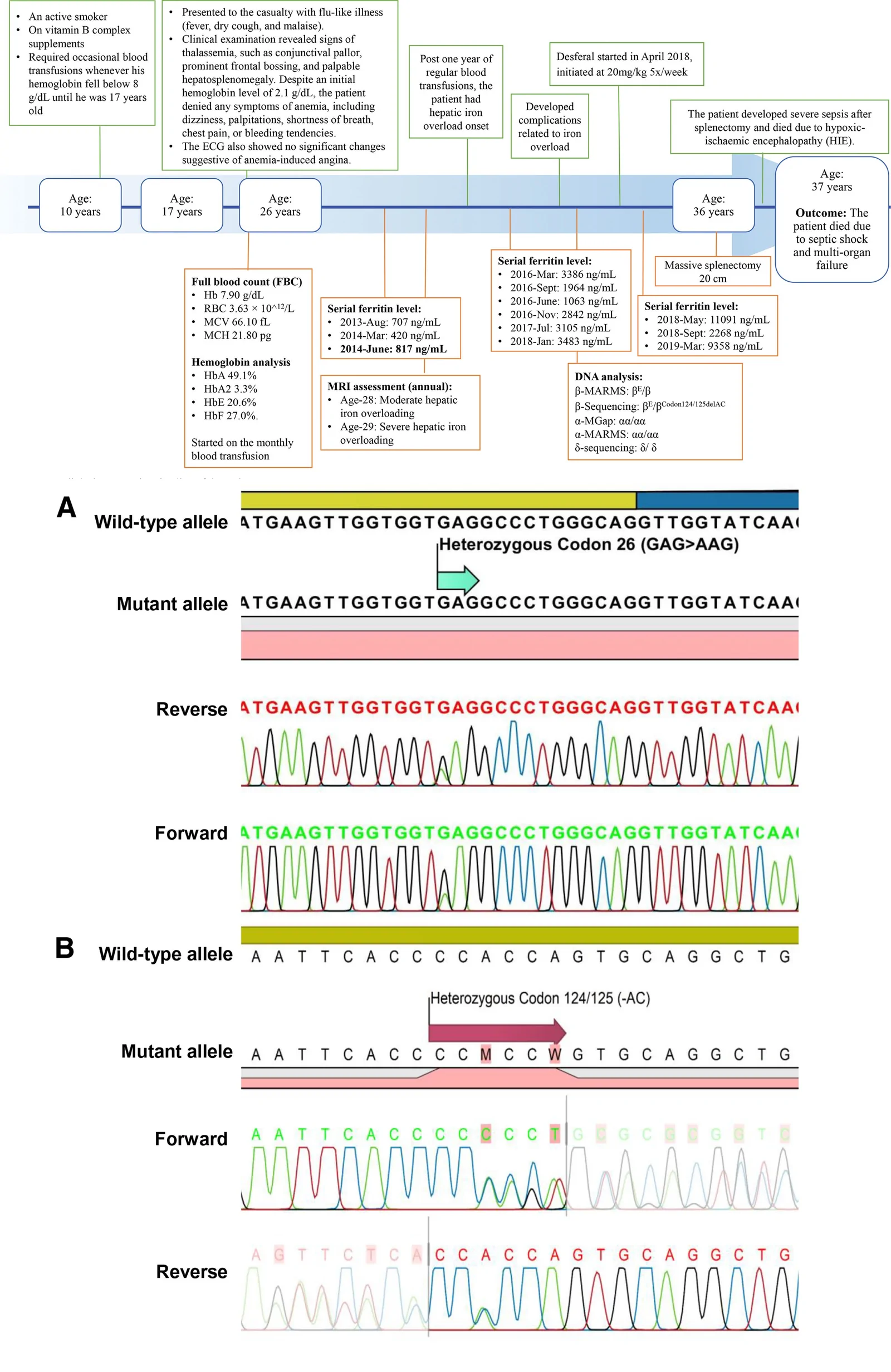
(A) Clinical presentation timeline of the patient. (B) Sanger sequencing chromatograms of the patient. (A) Identification of the heterozygous codon 26 (GAG > AAG) HbE mutation. (B) Identification of the novel codon 124/125 (‐AC) mutation. In both panels, the top bar represents the wild-type allele with reference sequence: yellow indicates the *HBB* transcript region and blue indicates the intronic region of the *HBB* gene. The second bar panel shows the consensus *HBB* gene sequence from the patient. The third and fourth panels displays sequencing chromatograms from the reverse and forward primers of the *HBB* gene respectively. Arrows indicate the reading frame direction for the codon 26 (GAG > AAG) and codon 124/125 (‐AC) sequences.

Desferal was initiated subcutaneously at 20 mg/kg 5 times weekly after persistent iron overload, with ferritin levels exceeding 10,000 ng/mL despite maximum tolerated oral Exjade, and MRI confirming severe liver iron deposition. According to Malaysian Clinical Practice Guidelines, cardiac MRI (T2*) and echocardiography are crucial for the early detection of cardiac siderosis and myocardial complications. However, the patient frequently missed many follow-up appointments, and only 2 MRI records (at ages 28 and 29) were available. This poor compliance ultimately contributed to the development of iron overload complications. At age 36, he presented with a massive spleenomegaly measuring 20 cm, necessitating splenectomy. He reported no pain 6 weeks postsurgery. No family history of thalassemia was noted. He died at age 37 due to septic shock and multi-organ failure. His clinical timeline is summarized in Figure 1A.

### 2.2. Molecular diagnosis

Based on the atypical hematological phenotype and presumptive diagnosis of HbE-BT, beta multiplex amplification refractory multiplex system-polymerase chain reaction was performed to detect common beta-thalassemia mutations. Analysis revealed that the patient was heterozygous for the codon 26 mutation (GAG > AAG; HbE). However, this single mutation did not fully explain the patient’s phenotype. Consequently, Sanger sequencing analysis was performed, which identified a second mutation: a 2-nucleotide deletion at codon 124/125. This confirmed a compound heterozygous state, with both the codon 26 (GAG > AAG) HbE variant and a novel mutation at codon 124/125 (‐AC) (HGVS: *HBB*:c.[79G>A];[375_376delAC]) (Fig. 1B). The novel mutation at codon 124/125 (‐AC) (HGVS: *HBB*:c.[79G>A];[375_376delAC]) has been submitted to the ITHANET database for public access and is available under the identifier IthaID: 4155 and IthaPhen PID: 4676. The complete molecular analysis findings, encompassing the *HBA1/2*, *HBB*, and *HBD* genes, are summarized in Table 1.

### 2.3. Predictive computational analysis of novel mutation

The *HBB*:c.375_376delAC variant was predicted to be “deleterious” by MutationTaster2021 and VEP, indicating high impact. MutationTaster2021 predicted a loss of beta helix (β125–β146) and a slightly truncated protein (missing <10% of amino acids), potentially leading to nonsense-mediated mRNA decay. Specifically, the frameshift mutation causes proline to serine at position 126 and introduces a premature stop at 139 (p.[P126Sfs*14]). This novel variant was notably absent from major population databases such as ExAC and gnomAD, as well as disease databases like HbVar. However, the data has since been submitted to the ITHANET database for public access and is available under the identifier IthaID: 4155. Following ACMG guidelines, both Franklin and VarSome classified it as “likely pathogenic,” primarily based on PVS1 and PM2 criteria. Furthermore, FATHMM linked *HBB*:c.375_376delAC to blood disorders, confirming its damaging nature. Structural analysis using SWISS-MODEL, coupled with validation tools like PROCHECK, VERIFY3D, and ERRAT, yielded high-quality models, with over 90% residues falling within allowed regions and scoring above established thresholds (Table 2). Comparison between the wild-type and mutant structures revealed significant alterations in amino acid interactions and overall protein conformation. Specifically, the p.(E27K) mutation, which replaces glutamic acid with lysine, disrupted existing hydrogen bonds while forming new interactions with His-117. The frameshift variant p.(P126Sfs*14) resulted in a truncated protein, profoundly impacting its secondary structure, particularly at the carboxyl terminus.

**Table 2:** Quality assessment of the predicted wild-type and mutant structures using SWISS-MODEL.

Wild-type/variant	PROCHECK (Ramachandran plot statistic [%])					VERIFY3D		ERRAT (%)
	Most favored	Additionally allowed	Generously allowed	Disallowed	*Z*-score		Overall quality factor score	
Wild-type	94.2	5.8	0	0	95.82		99.8145	
Variants: p.(E27K) and p.(P126Sfs*14)	93.3	6.7	0	0	89.75		99.0403	
Acceptance range	More than 90% indicating good quality structure					At least 80% of the amino acids have scored ≥0.1 in the 3D/1D profile		The accepted range for a high-quality model is >50%, and a value of 95% or higher is indicative of a good structure

## 3. Discussion

**Figure 2.**
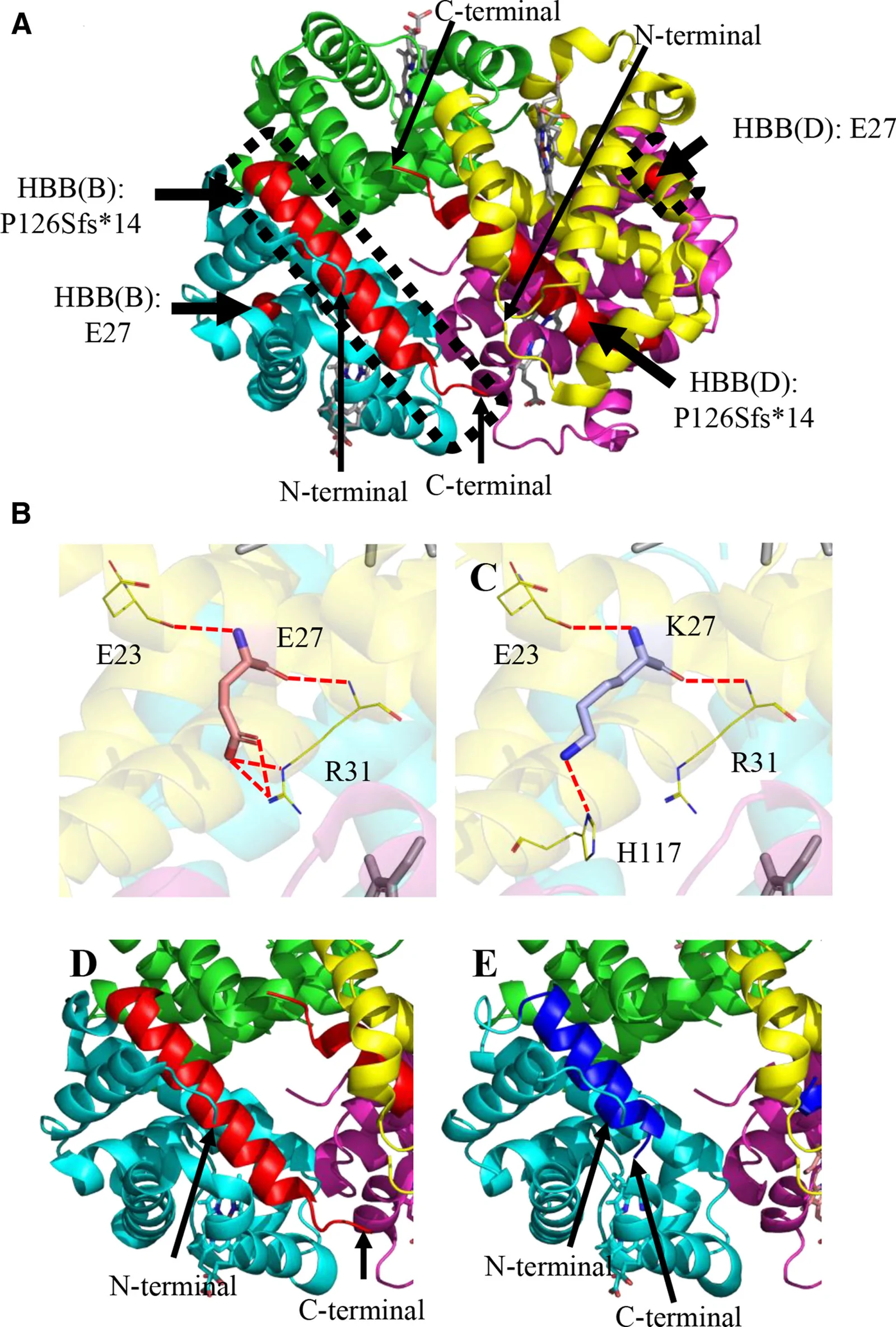
Models of hetero-tetramer wild-type HBA and mutant human HBB proteins. (A) Mutations mapped onto subunits B (cyan) and D (yellow) of the HBB protein. Wild-type HBA protein subunit A (green) and C (magenta) are also shown. Black arrows indicate the N-terminal, C-terminal, and mutant positions. Black dashed boxes highlight the positions of mutants p.(E27K) and p.(P126Sfs*14). (B) Structural models and hydrogen bond interactions of the wild-type glutamic acid residue (E27) with neighboring residues E23 and R31. (C) Structural model and hydrogen bond interactions of the mutant lysine (K27) with neighboring residues E23, R31, and H117. (D, E) Structural models comparing the wild-type beta-globin subunit (red alpha helix) and mutant beta-globin subunit (blue alpha helix) containing the frameshift variant p.(P126Sfs*14). The frameshift variant resulted in a substitution in the last 22 amino acid residues to a truncated 13-residue protein.

This study identifies and describes a novel *HBB*:c.375_376delAC mutation in a Malay male patient with HbE-BT intermedia, molecularly confirmed through Sanger sequencing. Predictive computational analyses consistently supported the deleterious impact of this variant, and structural analysis further enabled us to reconstruct the mutant protein and validated these predictions at the atomic level. Residue P126, located within the G-helix, is critical for overall maintaining protein structure; residues situated within helices typically play a vital role in stabilizing the helical conformation.^[10]^ Our 3D model illustrates that the p.(E27K) mutation alters the normal hydrogen bonds with neighboring charged residues (Fig. 2B and C). Additionally, the p.(P126Sfs*14) mutation results in a shortened alpha helix (Fig. 2D and E). Both changes could potentially influence the proper folding and stability of the G-helix. We, therefore, hypothesize that the compound heterozygosity of p.(E27K) and p.(P126Sfs*14) mutations collectively impairs normal hemoglobin function.

The p.(E27K) variant is well-established. Studies using the HbEKO transgenic mouse model have linked HbE expression to elevated reactive oxygen species and mild oxidative stress.^[11]^ This oxidative stress is further amplified in HbE-BT when HbE is combined with a beta-thalassemic allele.^[12]^ Several hemoglobin variants related to codons 124 and 125 have been reported (Hb Tunis, Hb Khartoum, Hb Ty Gard, Hb Tende, and Hb Novara) in the HbVar database. While most are clinically stable, Hb Khartoum, involving a proline to arginine substitution within the G-helix, has been associated with instability and severe neonatal jaundice in some cases.^[13]^ Hence, highlights the importance of this region for protein stability.

Frameshift mutations affecting codons 124 and 125 have also been reported in patients with dominant beta-thalassemia intermedia,^[14–17]^ leading to truncated beta-globin chains. A functional study of the *HBB*:c.375_376insCCAGT variant, which produces an elongated chain, demonstrated low levels of mutant *HBB* mRNA and an intermediate phenotype. Some research suggests that the mutant *HBB* mRNA, which generates a truncated beta-globin chain from nonsense and frameshift mutations, may account for a severe and dominant phenotype.^[18,19]^ Similarly, our patient, carrying the *HBB*:c.375_376delAC mutation is also predicted to produce a truncated beta-globin chain. The interaction between *HBB*:c.375_376delAC and HbE likely explains the intermediate phenotype observed, as well as the absence of any other detectable hemoglobin variant on protein electrophoresis, which aligns with phenotypes reported in HbVar.^[19]^ We were unable to determine whether the *HBB*:c.375_376delAC mutation was de novo or inherited, as parental samples were unavailable.

Initially, our patient did not require transfusions. However, after developing a high fever, ongoing transfusions became necessary. This change reflects the typical progression of intermediate-severity HbE-BT. Additionally, the patient’s case also illustrates the clinical challenge of maintaining consistent iron overload management. This difficulty, possibly compounded by uncontrolled and ineffective erythropoiesis, may have contributed to the changing transfusion needs. This case highlights the clinical significance of transfusion-dependent beta-thalassemia, especially the higher risk of morbidity and mortality from opportunistic infections following splenectomy. While vital for survival, managing transfusion-dependent beta-thalassemia long-term involves unique challenges, including a high risk of iron overload in patients requiring frequent transfusions, which can cause serious damage to the heart, liver, and endocrine glands. Despite chelation therapy, maintaining proper iron levels remains difficult, as shown by this patient’s severe hepatic iron overload. Splenectomy, often performed to lower transfusion needs and treat hypersplenism, adds other risks. Though it can improve quality of life and reduce transfusions, it significantly raises the risk of deadly infections, especially overwhelming postsplenectomy infection. The patient’s death from septic shock and multi-organ failure at 37, just 1 year after splenectomy at 36, underscores this serious postoperative danger.

## 4. Study strengths and limitations

Strengths: This report emphasizes the importance of comprehensive molecular analysis of thalassemia syndromes, particularly in regions where thalassemia carriers are common and genetically diverse. Identification of novel mutations is an important update to the mutation spectrum of HbE-BT and is crucial for understanding the genotype–phenotype relationship, prognosis, and burden of HbE-BT.

Limitations: No definitive clinical constraints were identified in the study. However, this case report was written posthumously. Most of the clinical information is based on electronic records. Notably, the patient is an active methamphetamine user, and the clinical history may not be completely accurate due to ongoing substance abuse.

## 5. Conclusion

In conclusion, this report describes a novel *HBB*:c.375_376delAC mutation that highlights the importance of a comprehensive diagnostic approach to thalassemia, particularly in ethnically diverse populations where a high degree of molecular heterogeneity is expected. The patient’s clinical course, characterized by an intermediate phenotype and later progression to transfusion dependence, underscores that relying solely on routine hematological parameters can lead to a delayed diagnosis and suboptimal management. Therefore, we advocate for the integration of advanced molecular diagnostics, such as sequencing, into the standard of care for patients with atypical or inconclusive screening results. This proactive approach is essential for accurate risk stratification, effective genetic counseling for family members, and the timely implementation of targeted management strategies to prevent life-threatening complications like iron overload and postsplenectomy sepsis.

## Acknowledgments

We thank the Director-General of Health, Ministry of Health (MOH) Malaysia, for the permission to publish this article.

## Author contributions

**Conceptualization:** Nur Aisyah Aziz, Ernie Zuraida Ali, Mohd Khairul Nizam Mohd Khalid, Wan Rohani Wan Taib.

**Data curation:** Nur Aisyah Aziz, Ernie Zuraida Ali, Faidatul Syazlin Abdul Hamid, Syahzuwan Hassan, Khairul Azhar Abu Bakar.

**Formal analysis:** Nur Aisyah Aziz, Ernie Zuraida Ali, Nursaedah Abdullah Aziz, Norodiyah Othman, Faidatul Syazlin Abdul Hamid, Syahzuwan Hassan.

**Funding acquisition:** Ezalia Esa, Wan Rohani Wan Taib.

**Investigation:** Nur Aisyah Aziz, Ernie Zuraida Ali, Mohd Khairul Nizam Mohd Khalid, Nursaedah Abdullah Aziz, Norodiyah Othman, Faidatul Syazlin Abdul Hamid, Syahzuwan Hassan, Khairul Azhar Abu Bakar.

**Methodology:** Nur Aisyah Aziz, Ernie Zuraida Ali, Mohd Khairul Nizam Mohd Khalid, Nursaedah Abdullah Aziz, Norodiyah Othman, Faidatul Syazlin Abdul Hamid, Syahzuwan Hassan.

**Software:** Nur Aisyah Aziz, Ernie Zuraida Ali, Nursaedah Abdullah Aziz, Faidatul Syazlin Abdul Hamid, Syahzuwan Hassan.

**Supervision:** Ezalia Esa, Wan Rohani Wan Taib.

**Writing – original draft:** Nur Aisyah Aziz, Ernie Zuraida Ali.

**Writing – review & editing:** Ernie Zuraida Ali, Mohd Khairul Nizam Mohd Khalid, Norodiyah Othman, Syahzuwan Hassan, Wan Rohani Wan Taib, Khairul Azhar Abu Bakar.
